# Balancing the risks of hydraulic failure and carbon starvation: a twig scale analysis in declining Scots pine

**DOI:** 10.1111/pce.12572

**Published:** 2015-06-27

**Authors:** Yann Salmon, José M. Torres‐Ruiz, Rafael Poyatos, Jordi Martinez‐Vilalta, Patrick Meir, Hervé Cochard, Maurizio Mencuccini

**Affiliations:** ^1^ School of GeosciencesUniversity of EdinburghEdinburghEH93JNUK; ^2^ BIOGECO, UMR 1202Université de BordeauxF‐33615PessacFrance; ^3^ UMR 1202 BIOGECOINRA33612CestasFrance; ^4^ Campus de UABCREAF08193BarcelonaSpain; ^5^ ICREACREAF08193BarcelonaSpain; ^6^ Universitat Autònoma de Barcelona08193Cerdanyola del VallèsSpain; ^7^ Research School of BiologyAustralian National UniversityACT 2601CanberraAustralian Capital TerritoryAustralia; ^8^ INRAUMR547 PIAFClermont UniversitéF‐63100Clermont‐FerrandFrance; ^9^ Present address: Department of PhysicsUniversity of HelsinkiPO Box 48Erik Palménin aukio 100014HelsinkiFinland

**Keywords:** drought, ecophysiology, leaf gas exchange, mortality, NSC, photosynthesis, transpiration, tree

## Abstract

Understanding physiological processes involved in drought‐induced mortality is important for predicting the future of forests and for modelling the carbon and water cycles. Recent research has highlighted the variable risks of carbon starvation and hydraulic failure in drought‐exposed trees. However, little is known about the specific responses of leaves and supporting twigs, despite their critical role in balancing carbon acquisition and water loss. Comparing healthy (non‐defoliated) and unhealthy (defoliated) Scots pine at the same site, we measured the physiological variables involved in regulating carbon and water resources. Defoliated trees showed different responses to summer drought compared with non‐defoliated trees. Defoliated trees maintained gas exchange while non‐defoliated trees reduced photosynthesis and transpiration during the drought period. At the branch scale, very few differences were observed in non‐structural carbohydrate concentrations between health classes. However, defoliated trees tended to have lower water potentials and smaller hydraulic safety margins. While non‐defoliated trees showed a typical response to drought for an isohydric species, the physiology appears to be driven in defoliated trees by the need to maintain carbon resources in twigs. These responses put defoliated trees at higher risk of branch hydraulic failure and help explain the interaction between carbon starvation and hydraulic failure in dying trees.

## Abstract 2

Understanding the physiological responses of leaves to drought is crucial since they are the site of both photosynthesis and transpiration, and hence play key roles in balancing the risks of carbon starvation and hydraulic failure. Co‐occurring healthy and unhealthy Scots pines showed different responses to summer drought: while healthy trees showed a typical response to drought for an isohydric species, atypical physiology in unhealthy trees appears to be driven by the need to maintain carbohydrate availability in needles and twigs. These responses put unhealthy trees at higher risk of branch hydraulic failure and help to explain the interaction between carbon‐starvation and hydraulic failure in dying trees.

## Introduction

Episodes of tree mortality in response to high temperature and extreme drought have been reported worldwide in the last few decades (e.g. Breshears *et al*. 2005; Allen *et al*. 2010; Peng *et al*. 2011; IPCC, 2014). Since forest species are adapted to their ecological niches, they are at risk when the intensity or length of drought events exceeds their specific limits of resistance. As future climate scenarios under ongoing climate change predict that drought frequencies and intensities will increase in several regions worldwide (Collins *et al*. 2013), the ability to understand, predict and model future tree response and survival to water deficit has become increasingly important.

Based on multiple observational and experimental studies, a theoretical framework has been developed in recent years proposing several mechanisms responsible for drought‐induced mortality (cf. recent review by McDowell *et al*. 2011): (1) C starvation (e.g. Galiano *et al*. 2011; Adams *et al*. 2013; Mitchell *et al*. 2013); (2) hydraulic failure of the plant water transport system (e.g. Anderegg & Anderegg 2013; Mitchell *et al*. 2013); and (3) phloem transport failure (e.g. Sala 2009; Adams *et al*. 2013; Hartmann *et al*. 2013; McDowell *et al*. 2013). In addition, biotic agents (insects, parasites and pathogens) may either make trees more susceptible to mortality by weakening the trees prior to drought stress or they may accelerate an ongoing decline process and eventually kill already weakened trees (e.g. Galiano *et al*. 2011; Zweifel *et al*. 2012; Gaylord *et al*. 2013; Oliva *et al*. 2014). However, the relative importance and interactions among these processes remain the source of ongoing debates (e.g. McDowell *et al*. 2008; McDowell & Sevanto 2010; Sala *et al*. 2010).

Leaves play a crucial role in balancing the risks of C starvation and hydraulic failure, since they are the site of C assimilation and sugar production, as well as the main source of water loss for trees. However, the role of needle and leaf physiology in drought‐induced tree mortality remains poorly understood, especially under field conditions. Several studies have focused on differences in gas exchange responses or hydraulic properties among different species. In particular, the comparison of drought‐sensitive and drought‐tolerant species (e.g. Mitchell *et al*. 2014) or of isohydric and anisohydric species (e.g. Limousin *et al*. 2013; Woodruff *et al*. 2015) under increasing water stress has helped gain insights into the role played by photosynthetic organs in the response to drought. A species‐specific trade‐off between drought resistance of leaf gas exchange and leaf physiological efficiency has been reported (Limousin *et al*. 2013), mirroring the trade‐off between xylem vulnerability and conductive efficiency (Tyree *et al*. 1994). However, this trade‐off does not seem to be ubiquitous (Limousin *et al*. 2013). Most studies report a gas exchange limitation under drought (Adams *et al*. 2013; Limousin *et al*. 2013), but little is known about differences in leaf physiology between healthy and non‐healthy trees within a single drought‐stressed population.

Scots pine (*Pinus sylvestris* L.) has become a model species to study physiological and demographic responses to drought (Jackson *et al*. 1995a, 1995b; Galiano *et al*. 2010, 2011; Zweifel *et al*. 2012; Poyatos *et al*. 2013), since it is a geographically widespread species (Critchfield & Little 1966) that grows under a large range of environments, thanks to its ability to adjust its hydraulic system to water availability. The main mechanisms of this adjustment involve tight stomatal control of transpiration and the regulation of the ratio between leaf and sapwood areas (*A*_L_ : *A*_S_, Mencuccini & Grace 1995; Poyatos *et al*. 2007; Martinez‐Vilalta *et al*. 2009). Nonetheless, mortality of Scots pine has been observed at the xeric border of its distribution in the Mediterranean area (e.g. Martinez‐Vilalta & Piñol 2002; Vilà‐Cabrera *et al*. 2013), central Turkey (Allen *et al*. 2010) and in dry alpine valleys (e.g. Bigler *et al*. 2007; Rigling *et al*. 2013), and this makes it a suitable model species to study the limits of physiological plasticity and adaptation to drought stress.

The physiological responses of Scots pine to drought are driven by its isohydric stomatal control (Irvine *et al*. 1998; Zweifel *et al*. 2007; Duursma *et al*. 2008; Poyatos *et al*. 2008). Under intense drought stress, further limitation of transpiring area in Scots pine is achieved by shedding needles (Kurkela *et al*. 2009), while chronic and extreme droughts further contribute to long‐term defoliation of Scots pine by impairing crown development (Thabeet *et al*. 2009). The decreased stomatal conductance leads to decreases in photosynthetic C assimilation and thus in non‐structural carbohydrate (NSC) availability under drought. Hence, under chronic drought, the isohydric strategy, together with a reduced canopy area, may be associated with an increased reliance on recent photoassimilates in defoliated trees and a possible decline in NSC storage (Eilmann *et al*. 2010), with the potential consequence of a higher risk of C starvation (McDowell *et al*. 2008; Breshears *et al*. 2009). Crown defoliation may thus render trees more vulnerable to subsequent droughts, and indeed, levels of crown defoliation have become a good indicator of the individual vigour of Scots pine under drought stress (Dobbertin *et al*. 2010; Galiano *et al*. 2010, 2011; Barba *et al*. 2013; Poyatos *et al*. 2013). In particular, high levels of defoliation (with a crown reduced to less than 50% of its original area) have been associated with reduced NSC availability and higher mortality (Galiano *et al*. 2011; Poyatos *et al*. 2013).

However, defoliated Scots pines also show higher sensitivity in soil‐to‐needle hydraulic conductance to drying soil, despite higher sap flow per needle area during non‐stressed conditions (Poyatos *et al*. 2013). This, therefore, suggests that defoliated trees may be more prone to hydraulic failure than non‐defoliated trees, despite the isohydric tendency of Scots pine. Xylem embolism caused by high tension during water transport (Tyree & Zimmermann 2002) can take place in any plant organ depending on its vulnerability to cavitation (Faustino *et al*. 2013), and may finally cause tree death due to reduced hydraulic efficiency of the plant (Tyree & Sperry 1988; McDowell *et al*. 2008). However, leaves and needles tend to have lower safety margins than stem xylem (Mayr & Cochard 2003; Bucci *et al*. 2013; Torres‐Ruiz *et al*. 2014) and can even act as hydraulic circuit breakers either via increased cavitation or conduit collapse (e.g. Zhang *et al*. 2014). Despite Scots pine being an isohydric species, needles might suffer cavitation during severe drought, which may impair their physiological response to stress.

To improve our understanding of the physiological responses of Scots pine needles to drought stress, we compared the responses of hydraulic and carbon‐related properties of defoliated and non‐defoliated trees over a growing season in a population of northern Spain that has experienced severe annual droughts since the 1990s and where tree mortality has previously been reported (Martinez‐Vilalta & Piñol 2002; Hereş *et al*. 2012). We measured diurnal courses of gas exchange by needles, as well as photosynthetic light response and CO_2_ response curves. We also determined the amount of NSC in needles and supporting twigs, the needle predawn and midday water potentials and their osmotic and turgor components. We combined needle transpiration and whole‐tree sap flow with water potentials (*Ψ*) to obtain twig‐ and tree‐level hydraulic conductance. Finally, we measured the twig morphological properties and determined the sensitivity of pine needles to embolism based on *Ψ*_50_ and *Ψ*_88_ (i.e. the water potentials at which 50 and 88% of the cumulative ultrasonic acoustic emissions are recorded). We hypothesized that: (1) defoliated trees show similar gas exchange performance to non‐defoliated trees (per unit leaf area); (2) defoliated trees have lower NSC reserves than non‐defoliated trees because of a lower biomass of photosynthetic tissues per twig; and (3) hydraulic properties and safety margins of needles remain unaffected by their level of defoliation.

## Materials and Methods

### Site and plant material

The study was conducted in north‐eastern Spain at the Poblet nature reserve in the Prades Mountains. The climate is Mediterranean, with a mean annual temperature of 11.3 °C (average for 1951–2010) and a mean annual rainfall of 664 mm, peaking in spring and autumn (cf. Ninyerola *et al*. 2007a, 2007b). The experimental area is similar to the one of Poyatos *et al*. (2013), and detailed information about the study site can be found in Hereter & Sánchez (1999). Briefly, the experimental site (41°19′58.05″N, 1°0′52.26″E; 1015 m asl) is located on a 35° northwest‐facing hill slope in the Tillar valley, on fractured schist, which results in fairly rocky and shallow (approximately 40 cm deep) xerochrept soils with a loamy texture and a high gravel content of approximately 44% (Barba *et al*. 2013).

The vegetation at the site is dominated by Scots pine [54% of the total basal area (BA) and mean diameter at breast height (DBH) of 0.32 m], and the evergreen holm oak (*Quercus ilex* L, 41% of the total BA and mean DBH of 0.15 m) is the main understorey species (Barba *et al*. 2013); further details on plot vegetation can be found in Poyatos *et al*. (2013). The Scots pine population is at least 150 years old and has remained unmanaged for the past 30 years (Hereş *et al*. 2012). The experimental site is located in an area where Scots pine is affected by drought‐induced die‐off with a standing mortality higher than 20%, and in which defoliated and non‐defoliated individuals co‐occur (Martinez‐Vilalta & Piñol 2002; Vilà‐Cabrera *et al*. 2013).

Eight non‐defoliated and eight defoliated Scots pines of fairly similar size (Supporting Information Table S1) were selected. Defoliation was expressed as the percentage of green needles, and was visually estimated relative to the healthy canopies of similar sized trees in the same population (Galiano *et al*. 2010). Individuals with 50% or fewer green needles were considered to be defoliated (Supporting Information Table S1). It should be noted that the term defoliation can be ambiguous as it also refers to active removal of leaf biomass by predatory agents; in the present study, it refers exclusively to the loss of green needle area compared with that of a similar tree growing under optimal conditions. This definition has been extensively used in the context of drought mortality studies (e.g. Bréda *et al*. 2006; Galiano *et al*. 2011; Poyatos *et al*. 2013).

Sampling of twig material for the determination of photosynthetic parameters, total water potentials, turgor and osmotic potentials and carbohydrate concentrations took place during three sampling campaigns during the growing season of 2012 (June, August and November) to represent the environmental conditions typical of warm and fresh springs, the peak of hot summer droughts and the cooler, more mesic autumns, respectively. Sampling of twigs for native levels of gas exchange did not take place in November 2012 because none of the branches within reach received any direct sunlight during the day.

### Meteorological and soil moisture measurements

**Figure 1:**
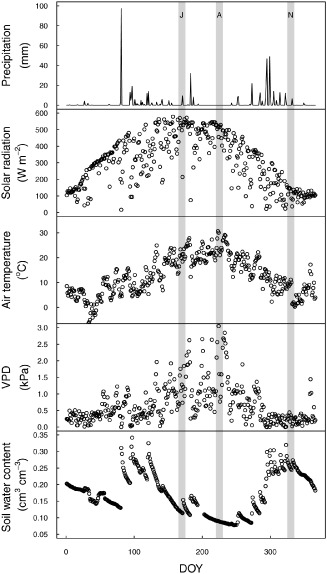
Meteorological conditions at the experimental site in 2012 as a function of day of the year (DOY): precipitation, solar radiation, air temperature, water vapour pressure deficit (VPD) and soil water content. The three grey areas represent the three sampling periods (J, A and N for June, August and November, respectively).

Meteorological and soil moisture measurements (Fig. 1) were performed using the same system as Poyatos *et al*. (2013). Briefly, a data acquisition system (CR1000 datalogger and AM16/32 multiplexers; Campbell Scientific Inc., Logan, UT, USA) was used to store 15 min averaged meteorological variables and soil moisture. Sensors for measuring air temperature and air relative humidity (CS215; Campbell Scientific Inc.), precipitation (52203; R.M. Young Company, Traverse City, MI, USA), total solar radiation (SP1110; Skye Instruments Ltd, Llandrindod Wells, Powys, UK) and wind speed (05103‐5; R.M. Young Company) were installed at the top of a 16‐m‐tall tower within 20 m of the plot centre. Average volumetric soil water content (SWC) in the upper 30 cm of soil was monitored using six frequency domain reflectometers (CS616; Campbell Scientific Inc.) randomly distributed within the plot, and raw measurements were corrected according to Poyatos *et al*. (2013). Water vapour pressure deficit (VPD) was calculated from air temperature and humidity (Fig. 1). To allow comparison of 2012 with other years, VPD and SWC are also presented for 2010, 2011 and 2013 (Supporting Information Fig. S1).

### Leaf gas exchange measurements

To determine *in situ* native rates of leaf gas exchange (i.e. transpiration rate *E*, stomatal conductance to water vapour in the light, *g*_s_, as well as CO_2_ assimilation rate, *A*_N_), gas exchange variables were measured every 2 h for 1 d on sun‐exposed, mid‐canopy twigs with fully expanded needles of three defoliated and three non‐defoliated trees. Water use efficiencies (WUEs) were calculated as the ratios between *A*_N_ and *E* and between *A*_N_ and *g*_s_. Measurements were carried out under ambient conditions with a portable photosynthesis system (Li‐6400XT; Li‐Cor Inc., Lincoln, NE, USA) equipped with a conifer chamber (6400‐22L; Li‐Cor Inc. The light source was removed to allow sunlight in the chamber through the transparent chamber lid). Note that due to the structure of the conifer chamber, gas exchange variables were measured at the scale of needles and supporting twigs. Foliage was left in the chamber until steady‐state gas exchange was reached (approximately 5 min), and results were then logged. Five measurements were averaged per tree, time and date.

To determine photosynthetic parameters, on each of the three sampling dates one sun‐exposed terminal twig was sampled with a pruning pole from the eight defoliated and eight non‐defoliated trees. To limit sampling effects on tree health, samples were shared between measurements of photosynthetic parameters and of water potential (see below). Immediately after sampling, the cut end of twigs was placed in water and recut to minimize embolism. Photosynthetic responses to *C*_i_, the sub‐stomatal CO_2_ concentration (referred to as CO_2_ response curves or *A–C*_i_ curves) and to light availability (light response curves) were measured with the same photosynthesis measurement system. The light intensity was controlled using a portable light source (6400‐22L; Li‐Cor Inc.) and the CO_2_ supply to the measurement chamber was controlled using the Li‐6400 mixer. Because of our sampling method (aimed at minimizing the effect of sampling on tree health), a delay between sampling and measuring occurred. However, measurements were performed no later than 48 h after sampling, with most of the samples measured in the first 24 h. To make sure the time between sampling and measurement had little influence on our results, gas exchange response curves were assessed for some randomly selected samples at the beginning and end of our measurement period (differences were typically around 5%; only *γ* – see below – varied by around 12%).

Each *A–C*_i_ curve was performed at a photosynthetically active radiation (PAR) intensity of 1000 *μ*mol_photon_ m^−2^ s^−1^ and at CO_2_ concentrations in the cuvette of 300, 200, 150, 125, 100, 75, 50, 40, 400, 400, 600, 900, 1250, 1500, 1750 and 2000 *μ*mol mol^−1^. A minimum of 3 min between steps was given to enable the needles to equilibrate to the new conditions. *A–C*_i_ curves were fitted according to Sharkey *et al*. (2007) using the following parameters: *V*c_max_, maximum carboxylation rate allowed by RuBisCO (ribulose 1·5‐bisphosphate carboxylase/oxygenase); *J*, rate of photosynthetic electron transport (based on NADPH requirement) at the measurement light intensity; *TPU*, triose phosphate use; *R*_d_, day respiration; and *g*_m_, mesophyll conductance. In this method, a non‐linear curve‐fitting method is used to estimate *g*_m_ and *R*_d_ by minimizing the sum of squared model deviations from the observed data based on both Rubisco‐limited (low internal CO_2_ concentration) and RuBP regeneration‐limited (intermediate to high internal CO_2_ concentration) portions of the *A–C_i_* curve (details and scripts are available at http://onlinelibrary.wiley.com/doi/10.1111/j.1365‐3040.2007.01710.x/).

Each light curve was performed at 400 *μ*mol mol^−1^ CO_2_ concentration and consisted of 18 PAR levels with measurements taken at 1850, 1500, 1000, 250, 100, 80, 60, 50, 45, 40, 35, 30, 25, 20, 15, 10, 5 and 0 *μ*mol_photon_ m^−2^ s^−1^. An additional measurement was taken at 0 *μ*mol_photon_ m^−2^ s^−1^ 10 min after the end of the light‐ curve to ensure that the dark respiration values were not influenced by light‐enhanced respiration. Light curves were fitted in *R* (R Core Team 2014) using the *nls* function to determine the parameters of a Mitscherlich equation [*A*_N_ = *A*_max_*(1 − exp(−*γ*(*PAR*‐*LCP*))), where *A*_max_ is the maximum photosynthetic rate (*μ*mol_CO2_ m^−2^ s^−1^), *PAR* the photosynthetically active radiation (*μ*mol m^−2^ s^−1^), *LCP* the light compensation point (*μ*mol m^−2^ s^−1^), and γ the apparent quantum yield or initial slope of the curve]. Day and dark respirations (*R*_day_ and *R*_dark_, respectively) were measured according to the Kok method (as described in Sharp *et al*. 1984).

Following these measurements, twigs used for gas exchange were sampled from the trees or from the branches used to measure the response curves, and the projected needle area was determined (Li‐3100C; Li‐Cor Inc.). Gas exchange values were recomputed with the exact needle area.

### Water potentials and hydraulic conductance

The water potentials *Ψ* of the terminal tips of branches – typically twigs around 10 cm long and their needles – were measured with a pressure chamber (PMS Instruments, Corvallis, OR, USA) at the same time as the gas exchange measurements in June, August and November. Measurements were taken both at predawn (*Ψ*_pd_; just before sunrise at 0300–0500 h, solar time) and at midday (*Ψ*_md_; 1100–1300 h, solar time). On each sampling date, one sun‐exposed terminal tip was sampled with a pruning pole from the eight defoliated and eight non‐defoliated trees. Prior to measurements, sampled tips were stored in humidified plastic bags to stop water loss from the twigs. The time between sampling and measurement in the field was typically less than 2 h.

To determine levels of osmotic adjustment in our sample trees, a subset of needles was placed in a large insulated box filled with dry ice for at least 2 h to lyse the cells. The needles were then placed in a syringe and pressed to extract the cell content. The solution obtained was analysed in an osmometer (Vapro 5520; Wecor Inc., Logan, UT, USA) to measure the osmotic component of the *Ψ*, later referred to as osmotic potential with the notation *Ψ*_md_o_ and *Ψ*_pd_o_ for midday and predawn, respectively. The turgor component of the *Ψ*, later referred to as turgor potential with the notations *Ψ*_md_t_ and *Ψ*_pd_t_ for midday and predawn, respectively, was calculated as the difference between *Ψ* and osmotic potential.

Twig level hydraulic conductance (*k*_twig_, mmol m^−2^ MPa^−1^ s^−1^) was calculated as: *k*_twig_ = *E*_md_/(*Ψ*_pd_ − *Ψ*_md_), where *E*_md_ is the transpiration rate (*E*) at midday. Whole‐tree hydraulic conductance (*k*_tree_, mmol m^−2^ MPa^−1^ s^−1^) was calculated as: *k*_tree_ = *J*_L,md_//(*Ψ*_pd_ − *Ψ*_md_), where *J*_L,md_ is the sap flow per unit needle area at midday (Supporting Information Fig. S2). Sap flow was measured during the three campaigns (June, August and November) on the same 16 trees employed to sample branches for gas exchange parameters and twig water potentials. Two 20‐mm‐long heat dissipation sensors (Granier 1985) were installed in the trunk at breast height, at opposite azimuths. Sap flow density, corrected for natural temperature gradients within the trunk and for radial variation within the xylem, was converted to sap flow per unit leaf area using tree sapwood depths and leaf areas. See Poyatos *et al*. (2013) for further details.

### Needle and twig NSCs

Twigs and needles from terminal tips of branches were sampled for NSC analyses at the same time as the terminal tips for *Ψ* (i.e. at predawn and midday in June, August and November). Bark was removed from twigs. All samples were microwaved for 180 s within 3 h of collection to stop enzymatic activity, oven‐dried for 72 h at 65 °C and ground to fine powder. Twigs and needles were separated and analysed independently. NSCs were defined as free sugars (glucose and fructose), low molecular weight sugars (free sugars and sucrose) plus starch, and were analysed according to Galiano *et al*. (2011) based on Hoch *et al*. (2002) with some minor modifications, and expressed as concentration (expressed in g of NSC per g of tissue and referred to as [NSC]).

### Morphological properties and vulnerability to cavitation

To determine the morphological properties of the twigs of our studied trees, one primary branch was harvested on four defoliated and four non‐defoliated trees in January 2013 and the annual shoot growth was measured for the last 3 years (i.e. 2010, 2011 and 2012). The number of needles per twig and per year and their projected area were measured with a standard desktop scanner. Twig mass was measured on 10 defoliated and 10 non‐defoliated trees in autumn 2014, making sure the sampled twigs were of equal age.

Vulnerability to cavitation of needles of defoliated and non‐defoliated trees was determined according to Charra‐Vaskou *et al*. (2012). Briefly, one additional primary branch was harvested on four defoliated and four non‐defoliated trees, sealed in dark plastic bags and maintained in contact with water overnight to allow full rehydration. One ultrasonic sensor (150 kHz resonance sensors, R15/C, 80–400 kHz) was attached to a group of 6–10 needles per branch to record ultrasonic emissions (UEs). Sensors were connected to a 20/40/60 dB preamplifier set to 40 dB and to an eight‐channel PCl‐2 system (PAC 125 18‐bit A/D, 3 kHz to 3 MHz PCl2; all components of the UE system from Physical Acoustics Corporation, Wolfegg, Germany). Water saturated branches were then allowed to dehydrate and the needle *Ψ* of 2–3 needles per branch was measured at different time intervals with a pressure chamber (Scholander type; PMS Instruments, Corvallis, OR, USA), while acoustic emission measurements were being made. Once the acoustic activity ceased, the cumulative number of UEs was calculated for each *Ψ* and vulnerability curves of percent cumulative ultrasonic emissions (PCUEs) were built accordingly. Curves were fitted using the equation provided by Pammenter & Van der Willigen (1998):

PCUE=100/(1+exp(a(Ψ−b))),

with *a* representing a dimensionless parameter controlling the shape of the curve and *b* the *Ψ* for a PCUE of 50% (i.e. *Ψ*_50_). These vulnerability curves enable the determination of the *Ψ* for a PCUE of 88% (*Ψ*_88_ *)* and, therefore, enable a comparison of the vulnerability to severe cavitation between defoliated and non‐defoliated trees.

### Statistical analyses

Statistical analyses (water potential and its components, [NSC] and its components, diurnal leaf gas exchange variables, light and *A–C*_i_ curve parameters) were performed in *R* (R Core Team 2014) using mixed effects models (package nlme; Pinheiro *et al*. 2013). These analyses employed dates and health status (defoliation level) as fixed factors and individual tree as a random factor. The interactions between the main factors were included in the initial model, and the model was simplified stepwise based on the corrected Akaike information criterion (AICc, for small sample sizes, package MuMIn; Barton 2014). In each step, we tested whether the simplified model was significantly worse than the original one using the likelihood ratio test.

Similarly, analyses of NSC and its components were carried out with organs (twig or needles) and time of sampling (predawn and midday) as fixed factors in addition to dates and health status, with their respective interactions also included in the initial model. Analyses of diurnal gas exchange variables (CO_2_ assimilation, stomatal conductance and transpiration) also included measurement hours, *VPD* (water VPD), *PAR* and air temperature (*T*_air_) as covariates to reduce noise from varying environmental conditions and help isolate the effects of the fixed factors (see Supporting Information Table S2). Diurnal gas exchange variables (CO_2_ assimilation, stomatal conductance and transpiration but not WUE) were square root transformed to reduce heteroscedasticity. Analyses of morphological variables were carried out overall and per year using a *t*‐test to compare defoliated and non‐defoliated trees.

## Results

### Branches morphology

**Figure 2:**
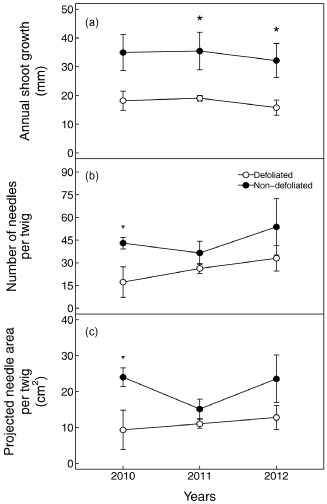
Annual shoot growth/length (a), number of needles (b) and needles' projected area (c) of defoliated (white) and non‐defoliated (black) Scots pine for the years 2010, 2011 and 2012. Each point represents the average value (*n* = 4) for a tree class. Error bars indicate ±1 SE. *Indicates significant differences between defoliated and non‐defoliated trees (*P* ≤ 0.05), while open triangle (∇) indicates marginal significances (*P* ≤ 0.1).

For the 3 years studied, defoliated trees showed lower annual shoot growth compared with non‐defoliated trees. In 2011 and 2012, defoliated trees had shoot growth of 19.1 ± 1.0 and 15.1 ± 2.6 mm, respectively, while twigs of non‐defoliated trees grew by 35.5 ± 6.5 and 32.1 ± 5.9 mm, respectively (Fig. 2a). This difference in growth was marginally significant in 2010. Twig biomass did not differ between defoliated and non‐defoliated trees (5.38 ± 1.20 and 4.95 ± 1.07 g, respectively). In addition, defoliated trees had a lower number of needles per year, with this difference being mostly due to the year 2010 (17 ± 10 versus 43 ± 8, respectively, Fig. 2b). Similar results were observed for the projected area of the needles, with lower area in defoliated than non‐defoliated pines (9.3 ± 5.5 cm^2^ versus 24.0 ± 2.6 cm^2^, respectively, Fig. 2c).

### Leaf gas exchange

**Figure 3:**
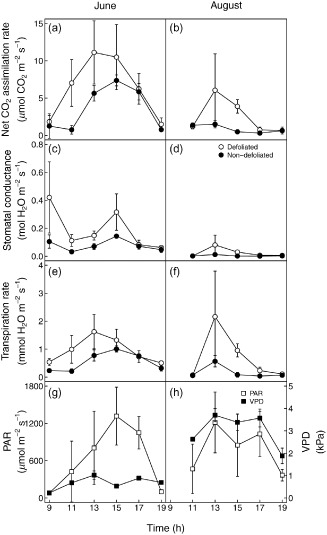
Diurnal changes in CO_2_ assimilation (a and b), stomatal conductance (c and d) and transpiration (e and f) in June (a, c and e) and August (b, d and f) in defoliated (white) and non‐defoliated (black) Scots pines. The variability of PAR and VPD values at times of measurements in June (g) and August (h) is also presented to allow interpretation of the variability in gas exchange. Each point represents the average value for a tree class at one date (*n* = 3). Error bars indicate ±1 SE. Symbols after the panel letter indicate significant (*, *P* ≤ 0.05) or marginally significant (∇, *P* ≤ 0.1) difference between defoliated and non‐defoliated trees in the panel. Differences are typically more significant if only the midday data are considered (i.e. first and last measurements excluded).

CO_2_ assimilation (*A*_N_, Fig. 3a,b), stomatal conductance (*g*_s_, Fig. 3c,d) and transpiration per unit leaf area (*E*, Fig. 3e,f) were significantly higher in defoliated than in non‐defoliated trees and in June than in August (note that short‐term differences in environmental conditions, and especially light and VPD, were accounted for by using them as covariates in the statistical analyses – see above and Supporting Information Table S2). Furthermore, *A*_N_, *g*_s_ and *E* were significantly affected by measurement time, showing an increase in the morning to early afternoon and a decrease in the mid to late afternoon. Interactions between health status, measurement dates and time were not significant in either *A*_N_, *g*_s_ or *E*. WUE (calculated as *A*_N_ */E*) was on average 8.44 ± 1.85 *μ*mol_CO2_ mmol_H2O_^−1^ and was only affected by measurement time, whereas WUE (calculated as *A*_N_ */g*_s_) was only affected by dates. Interestingly, neither of the two estimates of WUE was affected by the health status of the canopy but the *A*_N_ response to *g*_s_ was marginally higher (*P* = 0.08) in non‐defoliated trees than in defoliated trees in both June and August (Supporting Information Fig. S3). This would indicate that non‐defoliated trees tend to benefit more from opening their stomata than defoliated trees, although this result might be driven by the smaller range of *g*_s_ values experienced by non‐defoliated trees compared with defoliated ones.

Estimates of total daily twig scale assimilation (*A*_da_ *_y_* values and description in Supporting Information Table S3) were higher in defoliated than non‐defoliated trees, especially in August. Light response curve parameters (*A*_max_, *LCP* and *γ*) showed no significant differences between defoliated and non‐defoliated trees (Table 1). Model selection based on AICc resulted in final models with only health status and dates but no interaction for *A*_max_, *LCP* and *γ*. *A*_max_ and *γ* were also unaffected by dates, while *LCP* was lower in June compared with August and November. As expected, *R*_day_ was smaller than *R*_dark_ (by 30–60%; Table 1) and *R*_day_ was significantly affected by measurement date (*P* = 0.015), but defoliation class had no effect on either *R*_day_ or *R*_dark_.

**Table 1:** Variables (average ± SE, *n* = 7 or 8) from the light response curves and calculation of day and dark respirations according to the Kok method (*R*_day_ and *R*_dark_, respectively) of needles from defoliated (D) and non‐defoliated (ND) Scots pines in June, August and November

		June		August		November	
		D	ND	D	ND	D	ND
Light response curve	A_max_ (μmol_CO2_ m^−2^ s^−1^)	9.6 ± 2.4^a^	10.9 ± 0.3^a^	11.7 ± 1.8^a^	12.1 ± 4.8^a^	10.1 ± 0.7^a^	10.5 ± 2.3^a^
	LCP (μmol_photon_ m^−2^ s^−1^)	122.5 ± 19.0^a^	76.5 ± 69.2^a^	30.3 ± 23.3^a^	21.3 ± 20.4^a^	62.9 ± 9.7^a^	93.7 ± 19.2^a^
	*γ* (nmol_CO2_ μmol_photon_^−1^)	3 ± 0.1^a^	2.3 ± 1.0^a^	4.3 ± 0.6^a^	5.4 ± 1.8^a^	5.1 ± 0.7^a^	3.3 ± 0.5^a^
Kok method	R_day_ (μmol m^−2^ s^−1^)	2.52 ± 0.49^a^	2.93 ± 1.98^a^	1.13 ± 0.17^a^	1.80 ± 0.77^a^	2.02 ± 0.36^a^	2.49 ± 0.39^a^
	R_dark_ (μmol m^−2^ s^−1^)	4.02 ± 0.51^a^	4.47 ± 2.23^a^	2.89 ± 0.59^a^	3.62 ± 0.72^a^	3.36 ± 0.45^a^	3.51 ± 0.95^a^

Different letters indicate significant difference (*P* ≤ 0.05) between defoliated and non‐defoliated trees within a month. *A*_max_, maximum photosynthetic rate; *LCP*, light compensation point; *γ*, apparent quantum yield.

Results for *A*–*C*_i_ response curves were highly variable (Supporting Information Table S4), and this variability is difficult to reconcile with the comparatively more uniform results from the light response curves (Table 1). For example, in June, *V*_cmax_ and *J* in defoliated trees tended to be lower than in non‐defoliated trees, but both *V*_cmax_ and *J* increased from June to August in defoliated trees and decreased in non‐defoliated trees. As a result, *V*_cmax_ and *J* (and also TPU and *R*_d_) were marginally higher for defoliated trees in August. A post‐drought recovery in *V*_cmax_ and *J* was more evident in non‐defoliated trees in November (Supporting Information Table S4). These seasonal changes in needle photosynthetic apparatus may be partially explained by the interaction of drought responses and phenological maturation of needle tissues, as previously observed in species from drought‐stressed environments (e.g. in *Pinus halepensis*; Maseyk *et al*. 2008). However, methodological artefacts in *A*–*C*_i_ response curves cannot be totally excluded. Considering this and that *R*_d_ is only the residual of the *A*–*C*_i_ curve fitting, we focus later on *R*_day_ and *R*_dark_ calculated with the Kok method.

### Needles and twigs NSC

**Figure 4:**
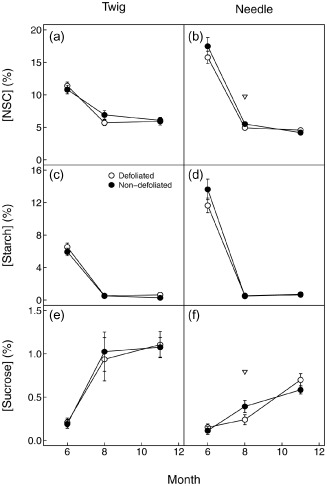
Seasonal changes in total non‐structural carbohydrates (a and b) and its components: starch (c and d) and sucrose (e and f) in defoliated (white) and non‐defoliated (black) Scots pine twigs (a, c and e) and needles (b, d and f). Samples taken at predawn and midday are pooled together, and consequently each point represents the average value for a tree type at one date (*n* = 14–16). Error bars indicate ±1 SE. Open triangles (∇) indicate marginal significant differences (*P* ≤ 0.1) between defoliated and non‐defoliated trees.

Defoliated trees had marginally significantly lower total [NSC] in summer (5.70 ± 0.45%) in needles compared with non‐defoliated trees (6.91 ± 0.67%, Fig. 4a,b); while no overall difference was observed between health classes. No significant differences were observed between defoliated and non‐defoliated needles and twigs for starch (Fig. 4c,d) and glucose + fructose (data not shown). Sucrose in needles was marginally lower in defoliated than non‐defoliated trees in summer and thus responsible for the marginally significantly difference in [NSC] observed between tree health classes (see above), but otherwise, no differences were observed between defoliated and non‐defoliated needles and twigs (Fig. 4e,f). However, defoliation status was kept in the final model selection based on AICc, except for sucrose. Further details on final model structure can be found in Supporting Information Table S5 and estimation of [NSC] per leaf area and total twig NSC are provided in Supporting Information Table S3.

Seasonally, total NSC and starch in both defoliated and non‐defoliated pines decreased from June to August (Fig. 4a,b for total NSC and Fig. 4c,d for starch). In August, values for starch were effectively zero for both organs and defoliation classes. For both defoliated and non‐defoliated trees, no recovery of total NSC or starch was observed from August to November. The high NSC and starch concentrations in June are probably temporary storage during the favourable months of spring to sustain aboveground and belowground sink activities during the stress period, but will not be further discussed due to the lack of sink organ data. On the contrary, sucrose increased from spring to autumn in both needles and twigs (Fig. 4e,f). Total NSC was significantly higher in needles than in twigs in spring (Fig. 4a,b), but no differences were observed later on. Glucose plus fructose concentration was higher in twigs (between 4 and 5.5%) than in needles (between 2.9 and 4.6%) and represented the main pool of low molecular weight sugars. In both organs, glucose and fructose concentrations were highest in summer. However, the variability over time of glucose and fructose concentration was much lower (∼1.5‐fold) than in the other NSC pools, namely starch (>10‐fold) and sucrose (>5‐fold).

When reported per needle area, estimates of [NSC] were similar, if not lower (see note in Supporting Information Table S3 legend), in defoliated than in non‐defoliated trees.

### Twig water potential components

**Figure 5:**
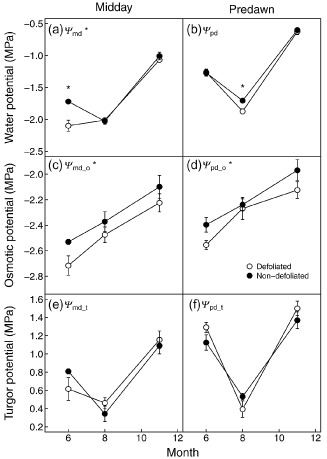
Seasonal changes in water potential (a and b) and its main components: osmotic potential (c and d) and turgor potential (e and f) in defoliated (white) and non‐defoliated (black) Scots pines at midday (a, c and e) and predawn (b, d and f). Each point represents the average value for a tree type at one date (*n* = 7 or 8). Error bars indicate ±1 SE. *Symbols after the panel letter indicate level of significance (*P* ≤ 0.05) between defoliated and non‐defoliated trees in the panel. * in the plots indicates significant differences (*P* ≤ 0.05) between defoliated and non‐defoliated trees at the considered date.

*Ψ*_md_ was significantly influenced by health status, sampling dates and their interaction. *Ψ*_md_ was lower in June in defoliated trees than in non‐defoliated trees (Fig. 5a; Supporting Information Table S6). In defoliated trees, *Ψ*_md_ did not change between June and August, while for non‐defoliated trees *Ψ*_md_ decreased to approximately −2.0 MPa in August, the same value observed for defoliated trees (Fig. 5a, Supporting Information Table S6). *Ψ*_md_ increased from August to November, reaching values of approximately −1 MPa for both types of trees (Fig. 5a). *Ψ*_pd_ tracked variations in soil moisture (Figs 1, 5b) and decreased from approximately −1.3 MPa in June to approximately −1.8 MPa in August, before increasing to approximately −0.6 MPa in November (Fig. 5b). No overall differences between defoliated and non‐defoliated trees were observed for *Ψ*_pd_; however, in August, *Ψ*_pd_ was significantly more negative in defoliated trees than in non‐defoliated trees (Fig. 5b; Supporting Information Table S6).

Both predawn and midday osmotic potentials (*Ψ*_md_o_ and *Ψ*_pd_o_) increased from June to August and from August to November in both defoliated and non‐defoliated trees (Fig. 5c,d). Defoliated trees had lower *Ψ*_md_o_ and *Ψ*_pd_o_ than non‐defoliated trees (Fig. 5c,d). Both predawn and midday turgor potentials (*Ψ*_md_t_ and *Ψ*_pd_t_) were also significantly different among sampling dates (*P* < 0.001). They first decreased from June to August and then increased from August to November in both defoliated and non‐defoliated trees (Fig. 5e,f). No overall significant differences were found between defoliated and non‐defoliated trees for either *Ψ*_md_t_ or *Ψ*_pd_t_.

*k*_twig_ increased fivefold between June and August in defoliated trees, while it decreased by approximately one‐third in non‐defoliated trees (Supporting Information Table S6). During the same period, *J*_L,md_ (Supporting Information Fig. S2) was higher during daytime and night‐time and showed later closure of the stomata during the day in defoliated trees, especially in August and November. As a result, *k*_tree_ remained stable in defoliated trees while it decreased by approximately three quarters in non‐defoliated trees. *k*_tree_ was significantly affected by date and also tended to be affected by the interactions between defoliation class and date. In June, both *k*_twig_ and *k*_tree_ were equal or higher in non‐defoliated than in defoliated trees, while in August both *k*_twig_ and *k*_tree_ were smaller in non‐defoliated than in defoliated trees. *k*_twig_ was higher than *k*_tree_ by a factor of 2 in June in both defoliated and non‐defoliated trees, while in August *k*_twig_ was 10 times higher than *k*_tree_ in defoliated trees and five times higher in non‐defoliated trees. Note that no statistical tests were performed on *k*_twig_ since these metrics were calculated using average values by defoliation class because of the different sampling designs for gas exchange, water potential and sap flow.

### Leaf xylem vulnerability to embolism

**Figure 6:**
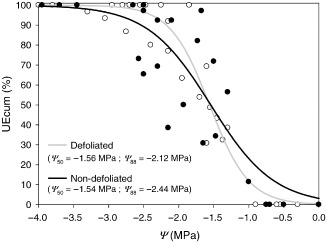
Xylem vulnerability curves of needles from defoliated (grey) and non‐defoliated (black) Scots pines represented as the percentage of cumulative number of ultrasonic emissions (UEcum) as a function of needle water potential (*ψ*). Values of the needle water potential inducing 50% (*Ψ*_50_) and 88% (*Ψ*_88_) of cumulative ultrasonic acoustic emissions from needles.

The curves of PCUEs obtained for the two health classes of trees were similar, with a *Ψ*_50_ at approximately −1.5 MPa in both cases (Fig. 6). However, non‐defoliated trees tended to reach a critical level of 88% PCUE at a slightly more negative needle *Ψ* than defoliated trees (Fig. 6), although the difference was not statistically significant.

## Discussion

The physiological responses to drought of needles from defoliated trees clearly differed from those from non‐defoliated trees. Contrary to our hypothesis, needles of defoliated trees maintained higher gas exchange rates during the drought stress period. This presumably allowed needles and terminal twigs of defoliated trees to maintain a similar [NSC] to those of non‐defoliated trees. However, the higher gas exchange rates during drought in defoliated trees came with increased risk of hydraulic failure in needles, because defoliated trees were slightly more sensitive to critical levels of needle embolism and were more water stressed (more negative water potentials) than non‐defoliated trees, especially during June at midday and August at predawn.

### Carbon assimilation and water loss

Despite having a smaller needle area per twig, defoliated trees showed higher *A*_N_ and *A*_day_ during the growing season than non‐defoliated trees. The latter showed a typical response to drought in Scots pines: decreased C assimilation during periods of low water availability (e.g. Berninger *et al*. 1996; Irvine *et al*. 1998). The unusual gas exchange response to water deficit in an isohydric species observed in the defoliated trees suggests that while non‐defoliated trees can afford a period of low C intake, defoliated trees need to maintain C assimilation during the period of peak stress. The higher *A*_N_ in defoliated trees came at a cost in terms of water loss because of higher *E* throughout the growing season. In addition, defoliated trees showed no improvement in WUE and smaller *A*_N_ gain per unit *g_s_* increase than non‐defoliated trees. This result is further supported by the higher sap flow per needle area, later closure of the stomata during the day and higher night‐time sap flow in defoliated trees, especially in August and November. Poyatos *et al*. (2013) observed similar sap flow patterns under favourable hydrological conditions in 2011. At the peak of the drought in 2011, both types of tree achieved very low sap flow rates, while in the present study higher sap flow was maintained in defoliated trees throughout the growing season. This is possibly due to the differences in drought stress development between years: 2011 had a mild but extremely long drought, while 2012 was a very hot and dry summer during which VPD and thus evaporative demand reached extreme values for the site (Supporting Information Fig. S1).

The differences in *E* between defoliated and non‐defoliated trees were associated with differences in the *g_s_* response to drought. Non‐defoliated trees closed stomata to limit water loss under drought, in agreement with existing knowledge of Scots pine needle physiology, thus exhibiting tight stomatal control in order to avoid critically low needle *Ψ* values (Irvine *et al*. 1998; Zweifel *et al*. 2007; Duursma *et al*. 2008; Poyatos *et al*. 2008). The *g_s_* decrease in non‐defoliated trees with decreasing water availability is also consistent with previous comparison of irrigated and non‐irrigated isohydric species, which showed gas exchange limitations under drought stress for both treatments albeit with a stronger limitation in non‐irrigated trees (e.g. Limousin *et al*. 2013; Torres‐Ruiz *et al*. 2013). Counter to expectation, the defoliated trees here showed a surprising stomatal response by maintaining *A_N_*, *E* and *g_s_* during the dry period; while their twig water potentials fell to values consistent with substantial losses of hydraulic conductance (see discussion about hydraulic risk).

### Carbon reserves and use

Despite their smaller number of needles and their smaller area, defoliated trees were able to maintain an almost identical [NSC] in needles and supporting twigs compared with non‐defoliated trees. The measured values of [starch] and [low molecular weight sugars], that is, 2–15%, are comparable to those reported for Scots pine needles under drought in a dry alpine valley of Austria (Gruber *et al*. 2012). However, a more defoliated population in the same forest at Prades (with an average 26% green leaves instead of 42% in the present study) had lower [NSC] in branches and leaves than in the non‐defoliated trees (Aguadé *et al*. 2015). Thus, at medium levels of defoliation (i.e. this study), higher values of *A*_N_ allow partially defoliated trees to maintain their terminal branch [NSC]. However, with increasing defoliation, the remaining needles do not appear to be sufficient to keep the branch [NSC] at a level similar to that of non‐defoliated trees. This explanation is further supported by the marginal difference observed in August in needle [NSC], which is consistent with the onset of a defoliation and drought effect on needle [NSC].

The [NSC] in needles and supporting twigs depends not only on C input (photosynthesis, see above) but also on C output (respiration, growth and export). Estimates of [NSC] concentrations per needle area were similar, if not lower, in defoliated than in non‐defoliated trees, despite higher *A*_day_, suggesting that an equal or larger fraction of the daily assimilated C is transferred to the supporting twigs. Total twig [NSC] decreased from June to August in both defoliated and non‐defoliated trees, but more in the former than in the latter (Supporting Information Table S3), supporting the hypothesis that higher gas exchange in defoliated trees is a response to lower NSC availability. Growth and growth respiration can be considered negligible at the three measurement dates because stopping growth is the first response of plants to water shortage (Sala *et al*. 2010) during drought‐stressed periods (June and August) when C uptake is limited due to stomatal closure, and because of phenological considerations (and lack of direct sunlight due to the north‐facing slope) in November. Thus, maintenance respiration is the main form of catabolism during our study and it does not differ between health classes. The lower *R*_day_ in August compared with June and November is probably related to the down‐regulation of processes with high C‐respiratory cost, for example, protein synthesis, turnover and growth (Gibon *et al*. 2009; Hummel *et al*. 2010) during the stress period.

A set of 21 trees including the present 16 trees showed significantly lower trunk [NSC] in defoliated than non‐defoliated trees (0.7 and 1.05%, respectively; Poyatos *et al*. 2013). Thus, despite the lack of differences in [NSC] at the needle and twig level in the studied population, differences in [NSC] in the stem were observed. This result is consistent with Norway spruce responses to drought, in which needle [NSC] did not differ between dying and surviving tress, although dying trees had significantly lower amount of NSC in their roots (Hartmann *et al*. 2013). More severely defoliated trees from the same population also showed significantly lower [NSC] in trunk and roots (Aguadé *et al*. 2015), in further agreement with an additional Scots pine population in Northern Spain (Galiano *et al*. 2011). Although the defoliated trees studied here were able to maintain enough NSC reserves in the needles and twigs, they could potentially suffer from C starvation in non‐photosynthetic organs as the result of two non‐mutually exclusive mechanisms: (1) the total amount of photosynthetic area does not permit enough NSC to be produced to maintain a C balance similar to that of the non‐defoliated trees (Galiano *et al*. 2011); and (2) phloem transport from needles to C‐sink organs might be impaired due to the competition for water between phloem transport and transpiration (Nikinmaa *et al*. 2013). Recent modelling of the same Scots pine population (Mencuccini *et al*., 2015 and in review) suggests that direct phloem failure by high viscosity is unlikely to play a role in long‐term mortality risks, as it would require extremely negative twig *Ψ* (Sevanto 2014; Sevanto *et al*. 2014). However, this does not rule out a temporary loss of phloem transport capacity in defoliated trees, as a result of loss of cell turgor during periods of very low twig *Ψ* and high *E*. Indeed, at midday, needle turgor was only 0.3–0.4 MPa, while *Ψ*_md_ averaged around −2.0 MPa. By comparison, values of −2.5 MPa in both defoliated and non‐defoliated trees were reported for *Ψ*_md_ during the longer drought of 2011 (Poyatos *et al*. 2013). Nonetheless, the relatively high *A*_N_ and *E* in defoliated trees, coupled with low absolute values of [NSC] in leaves and twigs, suggest that potential turgor impairment of phloem transport must be a temporary, not a permanent, phenomenon.

### Hydraulic risk

As needles from defoliated trees maintained *A_N_* at the cost of losing more water (Fig. 3 and Supporting Information Fig. S1), more negative *Ψ*_md_ values were reached in June. These low *Ψ*_md_ were maintained throughout the summer in defoliated trees, while non‐defoliated trees reached similar values only at the peak of drought in August. Summer *Ψ* values were similar to those reported from the same site in 2010 and 2011. However, even lower values were measured in mid‐October 2011 after an extraordinarily long drought (Poyatos *et al*. 2013). Hence, in the short intense drought of summer 2012 (Supporting Information Fig. S1), Scots pine maintained higher *Ψ* levels than under less intense, but longer, drought conditions. Between June and August in 2012, defoliated trees increased *k*_twig_ and kept *k*_tree_ constant, while both *k*_twig_ and, to a lesser extent, *k*_tree_ decreased in non‐defoliated trees. These results are superficially different from those in Poyatos *et al*. (2013), who observed a decrease in *k*_tree_ in both tree types between June and August in 2010 and 2011. This apparent discrepancy may be partly explained by the low soil moisture observed in June 2012 compared with other years (SWC in June 2012 is close to SWC usually measured later on in summer, Supporting Information Fig. S1). The high evaporative demand and the maintenance of gas exchange in summer 2012 in defoliated trees might have required adjustment of their hydraulic transport capacity. Liu *et al*. (2014) showed an association between experimental defoliation and increase in hydraulic conductance through changes in aquaporin expression. Although the causes of defoliation differ between the two studies (and thus the physiological mechanisms to respond to it are possibly different), changes in aquaporin expression may have played a role in the increase in *k*_twig_ in defoliated trees.

Additionally, defoliated trees tended to risk reaching extreme levels of embolism (88% PCUE, *Ψ*_88_) at higher (less negative) *Ψ* than non‐defoliated trees (Fig. 6). Values of *Ψ*_md_ measured in the field (Fig. 5) cannot be directly compared with needle xylem vulnerability curves obtained from PCUE (Fig. 6) because of uncertainties on the source of UE and of intra‐needle *Ψ* gradients. However, *Ψ*_md_ values measured in defoliated trees in June and August were close to the *Ψ*_88_ values in needles, suggesting a substantial risk of losses of leaf hydraulic conductance at midday, while *Ψ*_md_ in non‐defoliated trees decreased at the peak of the drought to values similar to those of defoliated trees, but without reaching *Ψ*_88_. Therefore, defoliated trees possibly operate within smaller safety margins than non‐defoliated trees, in agreement with similar observations from several angiosperm species, in which desiccated shoots had lower safety margins (*Ψ*‐*Ψ*_50_) than non‐desiccated ones (Nardini *et al*. 2013).

Hydraulic conductance and sap flow results also suggest a decoupling between whole‐tree sap flow and needle transpiration in defoliated trees during intense drought, probably mediated by water storage. The observations of a large increase in *k*_twig_ in defoliated trees in August, of differences in *Ψ*_pd_ between defoliated and non‐defoliated trees in August, and of a sustained higher nocturnal sap flow in defoliated trees in August and November all collectively suggest that defoliated trees may have been unable to restore capacitance reserves during the night to allow their needles *Ψ*_pd_ to equilibrate with the soil, and are consistent with the use of stored water to sustain transpiration during the day.

These adjustments of hydraulic conductance (and probably capacitance) allowed defoliated trees to successfully maintain their cell turgor at a level similar to that of non‐defoliated trees. This was however only achieved by maintaining more negative osmotic pressures through the day and through the year (i.e. accumulating higher solute concentrations). Based on the assumption that soluble sugars would be the only solute, we estimated that the lower osmotic potential in defoliated trees could cost approximately a 0.05–0.10 mol L^−1^ increase in soluble sugar concentration in defoliated trees compared with non‐defoliated trees (Supporting Information Table S7). Plants use other solutes (e.g. potassium, calcium, sodium, amino acids) in addition to NSC to maintain osmotic pressure (e.g. Talbott & Zeiger 1996). Although not measured in the present study, it is unlikely that defoliated trees have better access to those solutes than non‐defoliated trees since their acquisition is energy costly. Consequently, despite having similar [NSC], defoliated trees were likely to have less sugar available for physiological activity such as respiration, export to the sink organs, repair of damage, growth, defence and needle xylem reinforcement. In other words, in order to maintain needle cell integrity (i.e. turgor), defoliated trees needed to employ a higher fraction of their [NSC] for osmoregulatory purposes (Supporting Information Table S7) and were consequently at increased risk of C starvation.

## Conclusion

Our results offer new insights regarding the ongoing interactions between physiological processes leading to death in a Mediterranean Scots pine population. To understand how the present results generated insight for our interpretation of tree survival under drought, one should keep in mind that mortality happening at the branch scale negatively impacts the survival of the whole tree, while potential recovery of a branch (e.g. as measured by *Ψ*_pd_ and *Ψ*_md_ in autumn) does not necessarily translate into the recovery of the whole trees (i.e. other branches might have died). Defoliated and non‐defoliated trees differ in their carbon‐acquisition‐for‐water‐loss trade‐off characteristics. Defoliated trees increase carbon uptake under drought stress at the cost of higher water loss, while non‐defoliated trees do the opposite. Indeed, non‐defoliated trees showed a typical response for an isohydric species (i.e. closing stomata to maintain *Ψ* above critical levels), which, under the chronic drought conditions of the study site, may eventually lead to severe carbon deficits, but by doing so they increase their chance of survival to shorter and/or more intense drought by limiting the risk of hydraulic failure. In contrast, defoliated trees showed a less usual physiological response. Probably as a result of the smaller amount of photosynthetic tissue (leaf area) available, and the need to maintain C resources, they maintained higher gas exchange activity in the remaining needles. At moderate levels of defoliation (i.e. 35–50% of green leaves), defoliated trees maintained needle [NSC] similar to that of non‐defoliated trees, at least in twigs. However, it is unlikely that defoliated trees can maintain a favourable whole‐tree C balance, especially since they have to maintain more negative osmotic pressures to maintain cell turgor, a process which itself draws upon energy reserves. Overall, their smaller hydraulic safety margins and higher water losses also put them at higher risk of critical levels of embolism in terminal twigs and, therefore, expose them to higher chances of branch mortality by hydraulic failure. In turn, this results in further loss of photosynthetic tissues triggering a feedback loop between C starvation and hydraulic failure that increases the likelihood of subsequent death.

## Supporting information


**Figure S1.** Twenty‐day moving average of daytime VPD and soil water content. Whereas VPD peaked at about 1.3–1.45 in 2010–2011 and 2013, it reached approximately 2 at the end of August 2012 (DOY 239), and was already 1.48 on during the sampling period. This shows a persistently high evaporative demand condition during August 2012. The three grey areas represent the three sampling periods in 2012 (J, A and N for June, August and November, respectively).
**Figure S2.** Ten‐day means of hourly values of sap flow per unit leaf area (*J*_L,md_) from defoliated (red) and non‐defoliated (green) trees (*n* = 8) at the three sampling periods. Dashed lines represent ±1 SE. Results show higher sap flow rates in defoliated trees and a more gradual decline during the day, in agreement with transpiration data. The late morning increase, especially in November, is due to the north‐facing slope resulting in trees getting sunlight later in the morning than tree growing on flat surface.
**Figure S3.** *A*_N_ response to *g*_s_ in June and August in defoliated (white) and non‐defoliated (black) Scots pines. Each point represents the average value for a tree class at one measurement time of the day (*n* = 3), that is, the data are the same as the one presented in Fig. 3 panels A to D. Error bars indicate ±1 SE. Note the difference in x‐axis between panels A and B. Furthermore, note that conductance and assimilation rates for the defoliated trees in June at 9 h were not used in this plot due to their particular stomatal opening (Fig. 3). The fitted linear model included date and defoliation as fixed effects. Defoliated trees in June: *A*_N_ = 27.15*g*_s_ + 3.52 (*R* ^2^ = 0.51); non‐defoliated trees in June: *A*_N_ = 45.38*g*_s_ + 0.04 (*R* ^2^ = 0.37); defoliated trees in August: *A*_N_ = 69.41*g*_s_ + 0.75 (*R* ^2^ = 0.92) and non‐defoliated trees in August: *A*_N_ = 86.55*g*_s_ + 0.51 (*R* ^2^ = 0.55). Date had a significant effect on both the intercept and slope of the regression (*P* < 0.001 and *P* = 0.0014, respectively). Defoliation had a marginally significant effect on the slope of the regressions (*P* = 0.082).
**Table S1.** Morphometric characteristics of the study trees. Characteristic: tree health or defoliation levels: D, defoliated and ND, non‐defoliated; diameter at breast height (DBH); tree height and percent of green leaves: green leaves.
**Table S2.** Significance levels of the fixed factors and environmental covariates (VPD, PAR, *T*_air_) employed in the linear mixed effects analysis of diurnal gas exchange variables at three dates during the growing season of 2012. Model selection was based on AICc. *P*‐values are given where significant or marginally significant. ns means non‐significant. Excluded means that the variable was excluded from the final model.
**Table S3.** Estimates of NSC concentration per needle area, total NSC in twigs and daily twig C assimilation per needle area *A*_day_. *A*_day_ was estimated as the product of the average daytime assimilation values (from Fig. 3) multiplied by the daylight length and by the total needle area reported in Fig. 2. Note that these estimates are calculated from average dates and tree health level values and do not allow testing of significance for difference between treatments or dates.
**Table S4.** Parameters (average ± SE, *n* = 7 or 8) of the *A*–*C*_i_ response curves of twigs from defoliated (D) and non‐defoliated (ND) trees in June, August and November. *V*c_max_, maximum carboxylation rate allowed by Rubisco; *J*, rate of photosynthetic electron transport (based on NADPH requirement); *TPU*, triose phosphate use; *R*_d_, day respiration; *g*_m_, mesophyll conductance. Different letters indicate significant difference (*P* ≤ 0.05) between defoliated and non‐defoliated trees within a month, while different letter with a minus sign indicates marginally significant differences (*P* ≤ 0.1).
**Table S5.** Structures employed for the linear mixed effects models explaining changes in total NSC and its components (starch, sucrose, glucose + fructose) as a function of season, organ, health class of the trees, time of day and respective interactions. Model selection was based on AICc. *P*‐values are given where significant or marginally significant. ns means non‐significant. Excluded means that the variable was excluded from the final model.
**Table S6.** Midday transpiration (*E*_md_), midday and predawn water potentials (*Ψ*_md_ and *Ψ*_pd,_ respectively), twig hydraulic conductance (*k*_twig_), whole‐tree hydraulic conductance (*k*_tree_), as well as their ratio in June and August. D and ND stand for defoliated and non‐defoliated trees, respectively. The ratios between June and August values are also given in the final two columns for all variables. *k*_tree_ was significantly affected by dates (*P* = 0.024) and tended to be affected by the interactions between defoliation class and dates (*P* = 0.060). No statistical tests were performed for *k*_twig_, since it was calculated based on health class averaged data.
**Table S7.** Estimates of carbohydrate concentration (mol L^−1^) required to maintain osmotic pressure in needle cells. Estimates were calculated using the Morse equation by keeping the Van't Hoff factor equal to 1 (i.e. assuming all non‐electrolyte solutes). The calculations assume that values of osmotic potentials for the two health classes of trees derive entirely from carbohydrate concentrations. Defoliated trees required about 5–10% higher NSC concentrations in needles for osmotic regulation.
