# Supplementary material for: Balancing the risks of hydraulic failure and carbon starvation: a twig scale analysis in declining Scots pine
Source: Plant Cell Environ. 2015 Jun 27;38(12):2575–88. doi: 10.1111/pce.12572 (PMC4989476; doi:10.1111/pce.12572)
Supplement: Supplementary file 1 — Figure S1. Twenty‐day moving average of daytime VPD and soil water content. Whereas VPD peaked at about 1.3–1.45 in 2010–2011 and 2013, it reached approximately 2 at the end of August 2012 (DOY 239), and was already 1.48 on during the sampling period. This shows a persistently high evaporative demand condition during August 2012. The three grey areas represent the three sampling periods in 2012 (J, A and N for June, August and November, respectively). Figure S2. Ten‐day means of hourly values of sap flow per unit leaf area (J L,md) from defoliated (red) and non‐defoliated (green) trees (n = 8) at the three sampling periods. Dashed lines represent ±1 SE. Results show higher sap flow rates in defoliated trees and a more gradual decline during the day, in agreement with transpiration data. The late morning increase, especially in November, is due to the north‐facing slope resulting in trees getting sunlight later in the morning than tree growing on flat surface. Figure S3. A N response to g s in June and August in defoliated (white) and non‐defoliated (black) Scots pines. Each point represents the average value for a tree class at one measurement time of the day (n = 3), that is, the data are the same as the one presented in Fig. 3 panels A to D. Error bars indicate ±1 SE. Note the difference in x‐axis between panels A and B. Furthermore, note that conductance and assimilation rates for the defoliated trees in June at 9 h were not used in this plot due to their particular stomatal opening (Fig. 3). The fitted linear model included date and defoliation as fixed effects. Defoliated trees in June: A N = 27.15g s + 3.52 (R 2 = 0.51); non‐defoliated trees in June: A N = 45.38g s + 0.04 (R 2 = 0.37); defoliated trees in August: A N = 69.41g s + 0.75 (R 2 = 0.92) and non‐defoliated trees in August: A N = 86.55g s + 0.51 (R 2 = 0.55). Date had a significant effect on both the intercept and slope of the regression (P < 0.001 and P = 0.0014, respectively). Defoliation had a marg [file PCE-38-2575-s001.docx]

**Figure S1:** 20-day moving average of daytime VPD and soil water content. Whereas VPD peaked at about 1.3-1.45 in 2010-11 and 2013, it reached *ca*. 2 at the end of August 2012 (DOY 239), and was already 1.48 on during the sampling period. This shows a persistently high evaporative demand conditions during August 2012.The three grey areas represent the three sampling periods in 2012 (J, A and N for June, August and November, respectively).

**Figure S2:** 10–day means of hourly values of sap flow per unit leaf area (*J_L,md_*) from defoliated (red) and non-defoliated (green) trees (n=8) at the three sampling periods. Dashed lines represent ±1S.E. Results show higher sap flow rates in defoliated trees and a more gradual decline during the day, in agreement with transpiration data. The late morning increase, especially in November, is due to the North facing slope resulting in trees getting sunlight later in the morning than tree growing on flat surface.

**Figure S3:** *A_N_* response to *g_s_* in June and August in defoliated (white) and non-defoliated (black) Scots pines. Each point represents the average value for a tree class at one measurement time of the day (n=3), i.e., the data are the same as the one presented in Fig. 3 panels A to D. Error bars indicate ±1 SE. Note the difference in x-axis between panels A and B. Also note that conductance and assimilation rate for the defoliated trees in June at 9h were not used in this plot due to their particular stomatal opening (Fig. 3). The fitted linear model included date and defoliation as fixed effects. Defoliated trees in June: A_N_=27.15g_s_+3.52 (R^2^=0.51); Non-defoliated trees in June: A_N_=45.38g_s_+0.04 (R^2^=0.37); Defoliated trees in August: A_N_=69.41g_s_+0.75 (R^2^=0.92) and Non-defoliated trees in August: A_N_=86.55g_s_+0.51 (R^2^=0.55). Date had a significant effect on both the intercept and slope of the regression (p<0.001 and p=0.0014, respectively). Defoliation had a marginally significant effect on the slope of the regressions (p=0.082).

**Table S1:** Morphometric characteristics of the study trees. Characteristic: tree health or defoliation levels: D, defoliated and ND, non-defoliated; Diameter at breast height (DBH); tree height and percent of green leaves: Green Leaves

| Tree | Defoliation | DBH | Tree height | Green Leaves |
| --- | --- | --- | --- | --- |
|  |  | (cm) | (m) | (%) |
| 1 | D | 42.6 | 14.5 | 45 |
| 2 | D | 28.4 | 16.1 | 45 |
| 3 | D | 45.7 | 15.7 | 40 |
| 4 | D | 37.8 | 11.9 | 40 |
| 5 | ND | 35.5 | 16.1 | 80 |
| 6 | ND | 41 | 14.4 | 100 |
| 7 | ND | 44.7 | 17.3 | 100 |
| 8 | ND | 38.3 | 18 | 80 |
| 9 | D | 42.8 | 11.7 | 35 |
| 10 | D | 38.8 | 14.7 | 40 |
| 11 | D | 40.1 | 11.5 | 40 |
| 12 | D | 48 | 11.2 | 50 |
| 13 | ND | 28.5 | 11.2 | 55 |
| 14 | ND | 46.5 | 15.2 | 90 |
| 15 | ND | 26.7 | 8.2 | 80 |
| 16 | ND | 59.4 | 14.4 | 80 |

**Table S2:** Significance levels of the fixed factors and environmental covariates (VPD, PAR, T_air_) employed in the linear mixed effects analysis of diurnal gas-exchange variables at three dates during the growing season of 2012. Model selection was based on AICc. P-values are given where significant or marginally significant. ns means non-significant. Excluded means that the variable was excluded from the final model.

|  | A_N_ | g_s_ | E | WUE (A_N_/E) | WUE (A_N_/g_s_) |
| --- | --- | --- | --- | --- | --- |
| Date | <0.0001 | <0.0001 | <0.0001 | excluded | 0.0045 |
| VPD | ns | 0.0339 | ns | ns | excluded |
| PAR | <0.0001 | 0.0001 | <0.0001 | excluded | excluded |
| T_air_ | 0.0001 | 0.7421 | 0.0024 | ns | ns |
| Defoliation | 0.0197 | 0.0199 | 0.0073 | excluded | excluded |
| Time | <0.0001 | 0.0001 | <0.0001 | 0.0179 | excluded |
| Defoliation: Time | ns | 0.0798 | ns | excluded | excluded |

**Table S3:** Estimates of NSC concentration per needle area, total NSC in twigs and daily twig C-assimilation per needle area *A_day_*. *A_day_* was estimated as the product of the average daytime assimilation values (from Fig. 3) multiplied by the daylight length and by the total needle area reported in Fig. 2. Note that these estimates are calculated from average dates and tree health level values and do not allow testing of significance for difference between treatments or dates.

|  | June |  |  | August |  |
| --- | --- | --- | --- | --- | --- |
|  | Defoliated | Non-defoliated |  | Defoliated | Non-defoliated |
| [NSC]in needle per area (mg cm^-2^) | 5.1 | 6.0 |  | 1.6 | 1.9 |
| Total NSC in twigs (g) | 0.59 | 0.56 |  | 0.29 | 0.36 |
| A_day_ (umol cm^-2^ d^-1^) | 25.4 | 13.9 |  | 8.2 | 2.7 |

Despite the impossibility to test for significant differences in [NSC] per needle area between defoliated and non-defoliated trees, a qualitative comparison with the results presented in Fig. 4 helps to assess their importance. In both June and August estimates for NSC concentration per needle area in defoliated trees are 15% smaller than those for non-defoliated trees, while our measurements of NSC concentration per needle mass in defoliated trees are only 10% smaller than those of non-defoliated trees in both June and August.

**Table S4**: Parameters (average ± S.E., n=7 or 8) of the A-Ci response curves of twigs from defoliated (D) and non-defoliated (ND) trees in June, August and November. *V*c_max_, maximum carboxylation rate allowed by RuBisCO; *J*, rate of photosynthetic electron transport (based on NADPH requirement); *TPU*, triose phosphate use; *R*_d_, day respiration; and *g*_m_, mesophyll conductance. Different letters indicate significant difference (p≤0.05) between defoliated and non-defoliated trees within a month, while different letter with a minus sign indicates marginally significant differences (p≤0.1).

|  |  |  |  | June | | | | | | |  | August | | | | | | |  | November | | | | | | |
| --- | --- | --- | --- | --- | --- | --- | --- | --- | --- | --- | --- | --- | --- | --- | --- | --- | --- | --- | --- | --- | --- | --- | --- | --- | --- | --- |
|  |  |  |  | D | | |  | ND | | |  | D | | |  | ND | | |  | D | | |  | ND | | |
| CO_2_ response curve | Vc_max_ | (µmol _CO2_ m^-2^ s^-1^) |  | 49.3 | ± | 3.7^a^ |  | 95.5 | ± | 68.5^a^ |  | 105.2 | ± | 15^a-^ |  | 61.7 | ± | 12.4^b-^ | | 152.2 | ± | 12.1^a^ |  | 114.3 | ± | 8.8^b^ |
|  | J | (µmol _CO2_ m^-2^ s^-1^) |  | 75.8 | ± | 11^b-^ |  | 111.5 | ± | 8.5^a-^ |  | 93.5 | ± | 7^a^ |  | 67.3 | ± | 7.1^b^ |  | 100.3 | ± | 4.1^a^ |  | 95 | ± | 12.9^a^ |
|  | TPU | (µmol _CO2_ m^-2^ s^-1^) |  | 5.3 | ± | 1.1^a^ |  | 5.3 | ± | 4^a^ |  | 8.3 | ± | 0.5^a-^ |  | 6.3 | ± | 0.7^b-^ |  | 8.4 | ± | 0.3^a^ |  | 8.2 | ± | 1.2^a^ |
|  | R_d_ | (µmol m^-2^ s^-1^) |  | 2.7 | ± | 1^a^ |  | 2.8 | ± | 1.4^a^ |  | 6.3 | ± | 1.5^a-^ |  | 2.9 | ± | 0.8^b-^ |  | 8.4 | ± | 1.0^a^ |  | 6.3 | ± | 0.9^a^ |
|  | g_m_ | (µmol m^-2^ s^-1^ Pa^-1^) |  | 0.4 | ± | 0^a^ |  | 0.4 | ± | 0^a^ |  | 1.3 | ± | 0.5^a^ |  | 1.1 | ± | 0.2^a^ |  | 1.3 | ± | 0.3^a^ |  | 1.5 | ± | 0.3^a^ |

**Table S5:** Structures employed for the linear mixed effects models explaining changes in total NSC and its components (starch, sucrose, glucose + fructose) as a function of season, organ, health class of the trees, time of day and respective interactions. Model selection was based on AICc. P-values are given where significant or marginally significant. ns means non-significant. Excluded means that the variable was excluded from the final model.

| Variables | Total NSC | Starch | Sucrose | Glucose+Fructose |
| --- | --- | --- | --- | --- |
| Season | <0.0001 | <0.0001 | <0.0001 | 0.0001 |
| Organ | 0.0077 | <0.0001 | <0.0001 | <0.0001 |
| Health | excluded | ns | excluded | ns |
| Time | excluded | excluded | 0.012 | ns |
| Season:Organ | <0.0001 | <0.0001 | 0.0006 | 0.040 |
| Season:Health | excluded | ns | excluded | 0.06 |
| Season:Time | excluded | excluded | 0.002 | ns |
| Organ :Health | excluded | 0.08 | excluded | 0.07 |
| Organ :Time | excluded | excluded | 0.06 | ns |
| Health:Time | excluded | excluded | excluded | excluded |
| Season: Organ :Health | excluded | 0.09 | excluded | excluded |
| Season: Organ :Time | excluded | excluded | 0.47 | 0.08 |
| Season:Health:Time | excluded | excluded | excluded | excluded |
| Organ :Health:Time | excluded | excluded | excluded | excluded |

**Table S6:** Midday transpiration (E_md_), midday and predawn water potential (Ψ_md_, Ψ_pd,_ respectively), twig hydraulic conductance (k_twig_) , whole tree hydraulic conductance (k_tree_), as well as their ratio in June and. D and ND stand for defoliated and non-defoliated trees, respectively. The ratios between June and August values are also given in the final two columns for all variables. k_tree_ was significantly affected by dates (p=0.024) and tended to be affected by the interactions between defoliation class and dates (p=0.060). No statistical test were performed for k_twig_, since it was calculated based on health class averaged data.

|  |  | June | |  | August | |  | ratio August/June | |
| --- | --- | --- | --- | --- | --- | --- | --- | --- | --- |
| Variables |  | D | ND |  | D | ND |  | D | ND |
| E_md_ (mmol m^-2^ s^-1^) |  | 1.62 | 1.01 |  | 2.16 | 0.57 |  | 1.33 | 0.56 |
| Ψ_md_ (MPa) |  | -2.10 | -1.72 |  | -2.10 | -2.10 |  | 1.00 | 1.22 |
| Ψ_pd_ (MPa) |  | -1.27 | -1.27 |  | -1.88 | -1.71 |  | 1.48 | 1.35 |
| k_twig_ (mmol m^-2^ MPa^-1^ s^-1^) |  | 1.95 | 2.24 |  | 9.82 | 1.46 |  | 5.03 | 0.65 |
| k_tree_ (mmol m^-2^ MPa^-1^ s^-1^) |  | 1.01 | 1.08 |  | 0.96 | 0.28 |  | 0.95 | 0.26 |
| k_twig_/ k_tree_ |  | 1.9 | 2.1 |  | 10.2 | 5.2 |  |  |  |

**Table S7:** Estimates of carbohydrate concentration (mol.L^-1^) required to maintain osmotic pressure in needle cells. Estimates were calculated using the Morse equation by keeping the Van’t Hoff factor equal to one (i.e., assuming all non-electrolyte solutes). The calculations assume that values of osmotic potentials for the two health classes of trees derive entirely from carbohydrate concentrations. Defoliated trees required about 5 to 10% higher NSC concentrations in needles for osmotic regulation.

| Date | Defoliated | |  | Non-defoliated | |
| --- | --- | --- | --- | --- | --- |
|  | Pre-dawn | Mid-day |  | Pre-dawn | Mid-day |
| June | 0.106 | 0.11 |  | 0.1 | 0.102 |
| August | 0.094 | 0.1 |  | 0.093 | 0.095 |
| November | 0.088 | 0.089 |  | 0.081 | 0.084 |
